# Testicular schistosomiasis mimicking hydrocele in a child: a case report

**DOI:** 10.11604/pamj.2020.35.56.16322

**Published:** 2020-02-26

**Authors:** Olubanji Ajibola Oguntunde, Sylvester Ikhisemojie, Sandra Esse Sonusi, Adeyemi Oyebode, Biade Abdulkareem, Adekunbiola Aina Banjo

**Affiliations:** 1 Department of Anatomic and Molecular Pathology, College of Medicine, University of Lagos, Lagos, Nigeria; 2 Maternal and Child Health, Randle General Hospital, Lagos, Nigeria

**Keywords:** Schistosomiasis, testicular hydrocele, Nigeria

## Abstract

Schistosomiasis is a disease of profound public health importance worldwide. Testicular schistosomiasis (TS) is however still considered as a rare entity despite the burden of the disease. We report a case of a 9 year old male who presented with features suggestive of testicular hydrocele. The spermatic cord and testis were seen as thickened lesion on examination and a biopsy taken revealed calcified ova of *Schistosoma haematobium*. This is being reported to enhance increased suspicion amongst surgeons in cases of testicular masses within endemic settings like Nigeria.

## Introduction

Schistosomiasis is a tropical disease affecting communities mainly with poor sanitation and limited access to safe water [1]. It is estimated that an average of 90% of the over 200 million cases of human schistosomiasis is from sub-Saharan Africa [2]. This parasitic infection is caused mainly by *Schistosoma mansoni, Schistosoma haematobium* and *Schistosoma japonicum*. This disease affects different organs with variable symptomatology. Involvement of the urogenital system would commonly include the bladder and present with haematuria while haematochezia is usually observed if the large intestine is involved [3]. Testicular affectation albeit, very rare could present as a mass, hydrocele or infertility [3–5]. The proposed pathophysiologic mechanism for testicular schistosomiasis (TS) is a haematogenous spread of schistosomal eggs via venous communication between mesenteric vein and the internal spermatic vein [6]. The index patient is a 9 year old with pre-operative diagnosis of hydrocele but was noticed to have a thickened spermatic cord and testis at surgery. A biopsy was taken and it turned out to be schistosomiasis. Patient has since commenced prazinquantel. The aim of this case report is to heighten the suspicion of TS even with an innocuous presentation as that of hydrocele in a paediatric age group.

## Patient and observation

**Figure 1:**
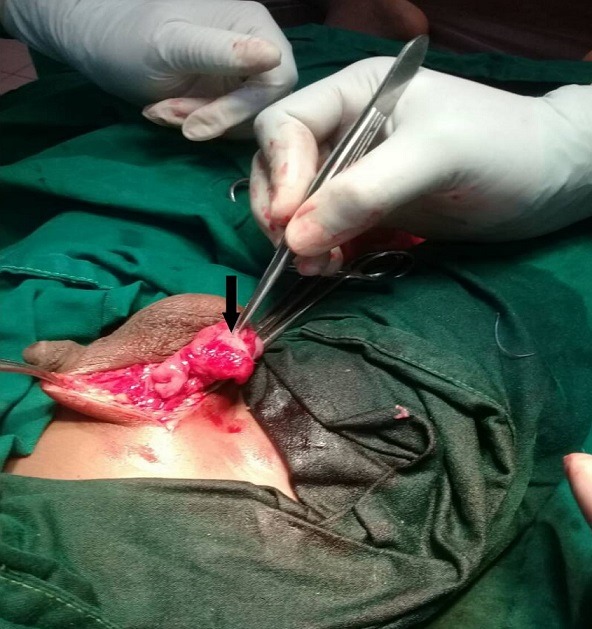
Showing thickened gubernaculum and testis

**Figure 2:**
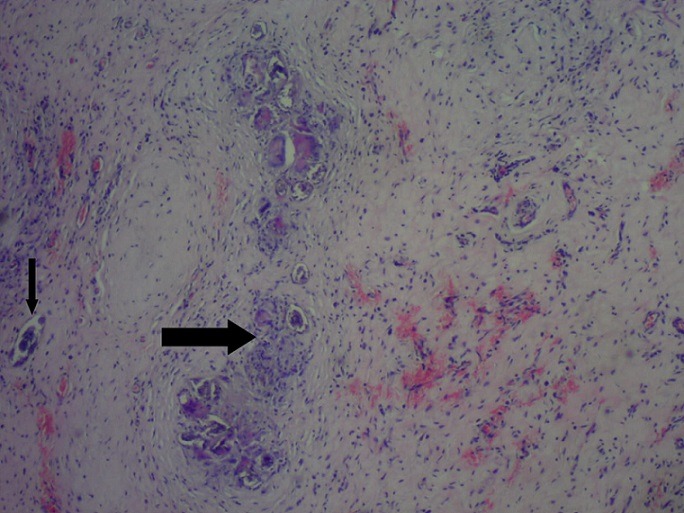
Showing granulomatous reaction and *Schistosoma haematobium* calcified eggs

**Figure 3:**
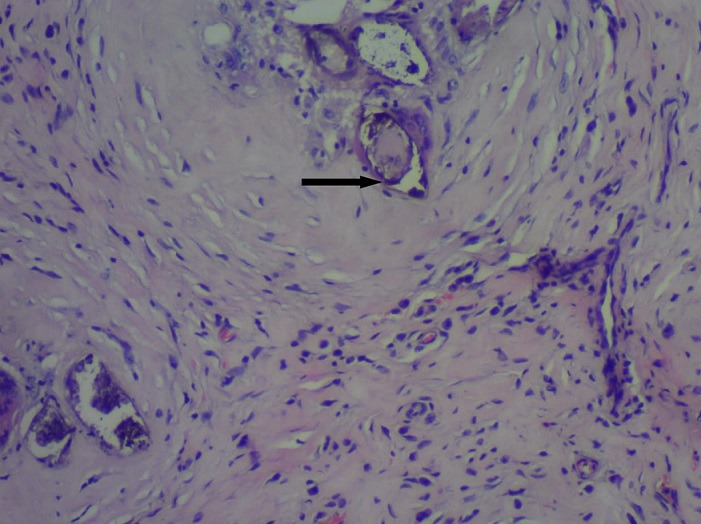
Showing the *Schistosoma haematobium* egg with terminal spine

A 9 year old male patient who was apparently well until about 10 months prior to presentation when he was observed to have developed a painless right scrotal swelling. The swelling steadily increased in size and never reduced. He was taken to a peripheral hospital where he was referred to the paediatric surgical unit of a secondary health care facility. He was born in Saki, a community in the south-western region of Nigeria where he grew up till about 3 years ago when he relocated with his family to Lagos metropolis. While in Saki, he often swam in the local river. There was neither history of haematuria in the past nor during the course of the illness. Examination revealed a small for age boy with an encysted, irreducible right hemiscrotal mass (10x4.5cm) which was neither tender nor warmer than the surrounding skin and trans-illuminated brilliantly to light. The left testis was unremarkable. A diagnosis of a right vaginal hydrocoele was made and the parent was counseled for surgery. Pre-operative laboratory investigations revealed a haematocrit level of 35%, HIV status (after obtaining consent from parents) was negative, HBsAg was positive, blood genotype AA, electrolyte, urea and creatinine values were essentially normal. He was subsequently scheduled for a day case routine herniotomy. Intraoperative findings include hydrocoele with multiple cysts, flat testis and thickened spermatic cord (Figure 1). Biopsy was taken from the testis and spermatic cord. He was discharged home on same day. Histology revealed fibrocollagenous tissue containing numerous granulomata in a background of dense admixed eosinophilic rich inflammatory infiltrates (Figure 2). Also identified were numerous multinucleated giant cells and schistosoma ova, some of which were calcified with terminal spine (Figure 3). The presence of terminal spine confirmed *Schistosoma haematobium* and a diagnosis of schistosomiasis was made. He was commenced on praziquantel 400mg stat following the histology report and he is being followed up in the outpatient clinic.

## Discussion

Schistosomiasis is a parasitic infection with the highest incidence in sub-Saharan Africa in Nigeria [2]. The risk factors include poor sanitation, lack of safe water, malnutrition and overcrowding [1]. The patient had positive history of swimming in the river and possible malnutrition as he appeared small for age at presentation which also puts him at further risk. He also spent the first 3 years of his life in Saki, a schistosoma endemic community in Oyo state which has an overall prevalence of 32.7% for *Schistosoma haematobium* infection amongst school children [7]. Some of the reported literatures on TS were also identified in endemic areas [3,4]. This emphasizes the importance of entertaining schistosomiasis as a differential diagnosis of testicular mass in patients who reside in endemic areas. Urinary bladder and the bowel are the commonly affected organs with classical presentations [3]. The diagnosis of schistosomiasis in many other organs are usually incidental findings on histology with no prior clinical suspicion [8, 9]. Such incidental findings have been described in the appendix, prostate, ovary, fallopian tubes and cervix. There are a few case reports on testicular involvement in adults and children.

Our case report showed a clinical presentation of an irreducible mass that is brilliantly trans-illuminable and characteristic of hydrocele with no symptom or sign to suspect schistosomiasis. The presentation in the index case concurs with the previous published studies in which no peculiar characteristic diagnostic clinical features of TS were reported [3, 4, 10]. The established pathogenesis is through the migration of larva from the lungs to the veins where the adult schistosoma lodge into the genitourinary venous plexus and the excretion of the eggs which subsequently elicit granulomatous inflammation. The hydrocoele can be explained by the granulomatous inflammation causing obstruction to the lymphatics in tunica and extravasation of the fluid transudates due to the ova. The definitive diagnosis of TS was demonstrated in our case report with evident granulomata surrounding the calcified schistosoma eggs in the specimen submitted. This is in agreement with previous reports on the diagnosis on TS [3, 4, 10]. The presence of terminal spine further characterized the specie in our case as typical of *Schistosoma haematobium*. Another lesson learnt in this case is to always submit specimens excised for histological analysis. If this case had passed unnoticed, there is a possibility of urinary bladder affectation which can give rise to squamous metaplasia, a precursor of squamous carcinoma of the bladder. Schistosomiasis is a medically treatable infectious disease. Praziquantel, 400mg stat as used in the index case is still the mainstay for treatment of schistosomiasis worldwide.

## Conclusion

Testicular schistosomiasis should always be considered in the differential diagnosis of testicular swelling. It is therefore important to ensure adequate clinical history; taking into consideration the endemic areas of schistosomiasis. Furthermore, suspicious areas at surgery should always be sent for histological analysis.

## Competing interests

The authors declare no competing interests.

## References

[1] Grimes JE, Croll D, Harrison WE, Utzinger J, Freeman MC, Templeton MR (2015). The roles of water, sanitation and hygiene in reducing schistosomiasis: a review. Parasites & vectors.

[2] Hotez PJ, Kamath A (2009). Neglected tropical diseases in sub-Saharan Africa: review of their prevalence, distribution, and disease burden. PLoS neglected tropical diseases.

[3] Rambau PF, Chandika A, Chalya PL, Jackson K (2011). Scrotal swelling and testicular atrophy due to schistosomiasis in a 9-year-old boy: a case report. Case reports in infectious diseases.

[4] Ekenze SO, Modekwe VO, Nzegwu MA, Ekpemo SC, Ezomike UO (2015). Testicular schistosomiasis mimicking malignancy in a child: a case report. Journal of tropical pediatrics.

[5] Adisa J, Egbujo EM, Yahaya BA, Echejoh G (2012). Primary infertility associated with schitosoma mansoni: a case report from the Jos plateau, north central Nigeria. African health sciences.

[6] Gryseels B, Polman K, Clerinx J, Kestens L (2006). Human schistosomiasis. The Lancet.

[7] Salawu AS, Asaolu SO, Sowemimo OA (2014). Co-infections with Schistosoma haematobium and soil-transmitted helminths among school-aged children in Saki, Oyo State, Nigeria. Journal of Public Health and Epidemiology.

[8] Mazigo HD, Giiti GC, Zinga M, Heukelbach J, Rambau P (2010). Schistosomal peritonitis secondary to perforated appendicitis. The Brazilian Journal of Infectious Diseases.

[9] Mazigo HD, Zinga M, Heukelbach J, Rambau P (2010). Case series of adenocarcinoma of the prostate associated with Schistosoma haematobium infection in Tanzania. Journal of global infectious diseases.

[10] Periyasamy P, Subramaniam SR, Rajalingham S (2011). An increasingly notorious mimicker of testicular tumours; crossing borders. BMJ case reports.

